# The Tumour Immune Microenvironment as a Predictor of the Response to Neoadjuvant Therapy in Rectal Cancer

**DOI:** 10.3390/cancers18081261

**Published:** 2026-04-16

**Authors:** Sreya Wadud, Eleanor J. Cheadle, Paul A. Sutton

**Affiliations:** 1 Colorectal and Peritoneal Oncology Centre, The Christie NHS Foundation Trust, Manchester M20 4BX, UK; sreya.wadud@student.manchester.ac.uk; 2 Targeted Therapy Group, Division of Cancer Sciences, University of Manchester, Manchester Academic Health Science Centre, Manchester M20 4BX, UK

**Keywords:** rectal cancer, tumour immune microenvironment, neoadjuvant therapy, tumour regression, pathological complete response

## Abstract

**Background**: Treatment response to neoadjuvant therapy in rectal cancer exhibits a considerable degree of interpatient heterogeneity. Select components of the tumour immune microenvironment have been identified as predictive biomarkers of therapeutic response, for which more evidence is required for future clinical prediction models. **Aim**: The research aimed to identify key tumour immune microenvironment biomarkers predictive of the response to neoadjuvant therapy through the systematic appraisal of existing literature. **Methods**: A structured search was performed across PubMed, Ovid Embase, and Cochrane databases to retrieve primary studies investigating the association between the tumour immune microenvironment and pathological complete response (pCR) or tumour regression grade (TRG) in patients with rectal cancer. Studies were screened against predefined inclusion and exclusion criteria. **Results**: Fifteen studies satisfied the inclusion criteria, with cohorts ranging between 24 and 298 participants with predominantly stage II–III disease. Considerable heterogeneity was observed in both types and methods of quantification of biomarkers. Biomarkers assessed in pretreatment biopsies included tumour-infiltrating lymphocytes (TILs), investigated by subtype (cluster of differentiation (CD)8+, CD4+, forkhead box protein 3+ (FOXP3)) or as a composite measure, as well as programmed death-ligand 1 (PD-L1), PD-1+, natural killer (NK) cells, CD163+, and CD68+. Findings showed that high densities of TILs—particularly the CD8+ subtype—consistently correlated with improved tumour regression. FOXP3+ and CD163+ were inconsistently associated with reduced treatment response. NK cells and CD68+ cells were less frequently investigated and yielded non-significant findings. **Conclusions**: CD8+ TILs have the potential to serve as predictive biomarkers of therapeutic response to neoadjuvant treatment in patients with rectal cancer. Inconsistent findings with FOXP3+ Tregs and CD163+ macrophages reinforce the need for their further investigation.

## Simple Summary

Treatment of rectal cancer frequently involves neoadjuvant therapy, aiming to downstage the tumour prior to surgical excision. The extent of tumour regression is highly variable, ranging from limited regression to pathological complete response (pCR), defined as the absence of any viable tumour tissue. With no reliable predictive model to risk-stratify tumour regression in patients with rectal cancer undergoing treatment, developing a method to identify candidates who are likely to achieve pCR would potentially improve rates of organ preservation. Emerging evidence has proposed components of the tumour immune microenvironment in mediating response to neoadjuvant therapy. The aim of this review was to identify potential immune biomarkers within rectal tumours predictive of the response to neoadjuvant therapy. We reviewed 15 retrospective/prospective cohort studies assessing the relationship between pretreatment tumour immune biomarkers and tumour regression through matched pretreatment and post-excision histological analysis. Our analysis identified a correlation between CD8+ tumour-infiltrating lymphocytes and tumour regression, thereby functioning as a potential predictor of treatment response. Furthermore, this review summarises the in vitro and translational studies in this emerging field.

## 1. Introduction

The current standard of care for locally advanced rectal cancer (LARC) involves neoadjuvant treatment (with either long-course chemoradiotherapy [nCRT], short-course radiotherapy, or total neoadjuvant therapy [TNT]) followed by total mesorectal excision. The aim of neoadjuvant treatment is to downstage the primary tumour prior to resection, reduce the risk of local recurrence, and increase the rate of sphincter/organ preservation [1]. The response to neoadjuvant treatment exhibits considerable interpatient heterogeneity, ranging from minimal tumour regression to the complete absence of viable tumour cells, defined as pathological complete response (pCR). Although rates vary with treatment modality, recent studies have demonstrated that almost a third of patients achieve pCR prior to surgical resection, thus opening up the possibility of non-operative management [2]. Omission of total mesorectal excision avoids potential postoperative complications, such as urinary and/or sexual dysfunction, and stoma formation [3,4]. However, without an established prediction model to identify patients likely to achieve pCR, patients are managed with a universal strategy, resulting in potential overtreatment and missed opportunities for organ preservation. Therefore, to deliver individualised care, it is imperative to identify factors that can reliably predict pCR.

A number of clinical and radiological factors have been evaluated with respect to their determination of therapeutic response. These include tumour size, TNM stage, radiation dose, and the time elapsed between completion of neoadjuvant treatment and surgery [5]. Within the past decade, the tumour immune microenvironment (TIME)—the ecosystem of immune cells and molecules within tumours which influence tumour biology [5]—has emerged as a potential mediator of tumour response to neoadjuvant treatment. Extensive research highlighting the importance of the TIME in other cancer types, most notably melanoma and non-small cell lung cancer [6,7,8], provides a strong rationale for investigating its significance in rectal cancer.

Cohort studies employing immunohistochemistry (IHC) on pretreatment biopsies appear to be the most common approach to explore this concept. Currently, the most commonly investigated components of the TIME in rectal cancer include CD8+ T cells, CD3+ T cells [9], and tumour-associated macrophages [10]. Whilst the majority of studies correlate high CD8+ densities with enhanced tumour regression, some show no association with response. Furthermore, the predictive value of FOXP3+ T regulatory cells (Tregs) and CD3+ remains controversial [9,11,12,13]. For example, some studies report high FOXP3+ density as a predictor of poorer tumour regression [14], whilst others report no significant correlation [9,14] or a positive association [12] despite using closely matched methodologies. The discrepancy may be attributed to the heterogeneity in outcome definition (i.e., pCR vs. TRG) or the different neoadjuvant regimens used.

In the absence of a reliable prediction model for rectal cancer, we conducted a review to critically appraise the current literature on the TIME, to identify potential and understudied biomarkers predictive of response to neoadjuvant therapy.

## 2. Methods

### 2.1. Review Protocol

This review was registered in the International Prospective Register of Systematic Reviews (PROSPERO), with identification number CRD420251073243, on 11 January 2025.

### 2.2. Search Strategy

A structured search was conducted on 2 June 2025 across the PubMed, Embase (via Ovid), and Cochrane Library databases for literature investigating the potential relationship between the TIME and response to nCRT or TNT. A search string was assembled using a combination of controlled vocabulary (i.e., MeSH terms, Emtree terms) and free text keywords derived from the PICO framework. Search terms included “rectal neoplasms”, “rectal cancer”, “rectal carcinoma”, “rectal adenocarcinoma”, “neoadjuvant therapy”, “chemoradiotherapy”, “total neoadjuvant therapy”, “induction chemotherapy”, “consolidation chemotherapy”, “tumour microenvironment”, “tumour infiltrating lymphocytes”, “tumour immune microenvironment”, “immune landscape”, “immune infiltration”, “pathologic complete response”, “tumour regression grade”, linked by Boolean operators “OR” and “AND” exclusively. Full details are found in Tables S1–S3.

A manual screening of reference lists was performed during full-text review to identify relevant articles not captured by the primary search strategy. These studies were equally subject to the same eligibility criteria and were only included if all criteria were satisfied. This review was performed and reported in concordance with the Preferred Reporting Items for Systematic Reviews and Meta-Analyses (PRISMA) guidelines (Supplementary File S1) [15].

### 2.3. Study Selection

Studies retrieved across the databases were pooled into Covidence (Veritas Health Innovation, Melbourne, Australia). Any articles identified more than once, for example, due to overlap between databases or having fulfilled both search strings, had their duplicates removed during the automatic screening process. Screening was performed by one reviewer and verified by a second. Unrelated papers were manually excluded based on title and abstract, and the remaining articles were reviewed in full against the following predetermined inclusion and exclusion criteria.

### 2.4. Inclusion and Exclusion Criteria

Full-text articles in English were eligible for inclusion. Only peer-reviewed primary research articles were included in the final selection. This review did not consult grey literature (e.g., conference proceedings, poster abstracts). No date restrictions were applied.

Studies eligible for inclusion must have assessed adult patients (18 years or older) diagnosed with rectal cancer of any stage by MRI, receiving either standard nCRT or TNT prior to planned total mesorectal excision. TNT was defined as the exclusively preoperative administration of systemic chemotherapy in conjunction with chemoradiotherapy. No restrictions were placed on the type of neoadjuvant regimen (i.e., long-course or short-course; induction vs. consolidation), the type of cytotoxic agent(s) used, or the interval between completion of neoadjuvant treatment and surgical resection. Studies involving the use of concomitant immunotherapy were excluded due to confounding alteration of the TIME profile prior to resection.

Additionally, studies must have assessed TIME-related immune biomarkers through analysis of pretreatment tissue biopsies of the primary rectal tumour. No restrictions were applied to the method of analysis, type of antibodies used, or the metrics used to quantify TIME components. Example parameters may include, but are not limited to, immune cell density, cell counts per high power field, or staining intensity. Studies that reported pre- and post-treatment changes were only included if they provided extractable data specific to the pretreatment immune profile. Articles exclusively assessing peripheral immune composition, such as through flow cytometry, were not considered representative of the TIME and therefore excluded.

Eligible studies must have additionally reported data quantifying the immediate response to neoadjuvant treatment in the form of either pathological complete response (pCR) rate, or a standardised criteria for the grading of tumour regression (TRG) either radiologically or histopathologically [16,17].

Only studies that satisfied all the above criteria were included in the final selection of articles.

### 2.5. Risk of Bias Assessment

Articles within the final selection were individually assessed by a single assessor and checked by a second for risk of bias using the Quality in Prognostic Studies (QUIPS) tool [18]. This tool considers six domains: study participation, study attrition, prognostic factor measurement, outcome measurement, study confounding, and statistical analysis and reporting, in order to quantify bias in non-randomised prognostic studies.

### 2.6. Data Extraction and Synthesis

Data was extracted from included studies by one reviewer, and independently checked by a second reviewer. Extracted data included patient demographics, NAT regimes, methods of assessing the tumour immune microenvironment, and associations with treatment response. No formal data synthesis was planned due to the expected levels of heterogeneity in patient cohorts, treatments received, translational analyses performed, and outcomes measured.

## 3. Results

### 3.1. Study and Patient Characteristics

**Figure 1:**
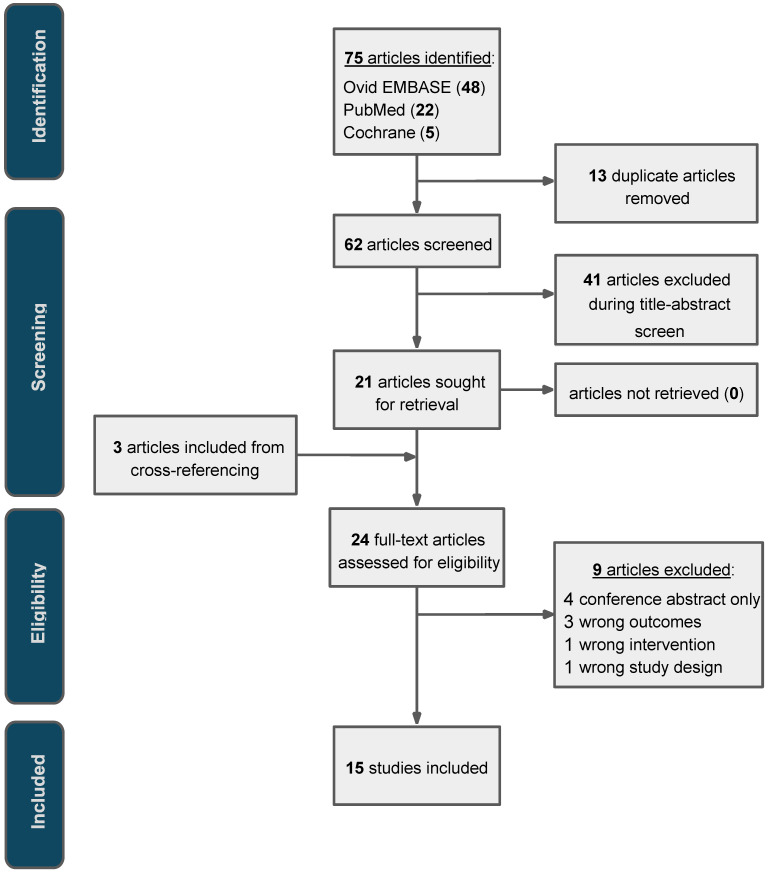
PRISMA flow diagram of study selection outlines the number of studies at each stage of the selection process, from identification, screening, and eligibility assessment, to final inclusion. Excluded papers are highlighted in Table S4.

A review of the literature on baseline TIME characteristics predictive of response to neoadjuvant treatment in locally advanced rectal cancer found a total of 15 articles satisfying the criteria of the search (Figure 1). A total of 2356 patients were investigated across 15 studies, and characteristics of the included studies are shown in Table 1. Study designs were predominantly retrospective (*n* = 11), with cohort sizes ranging from 24 to 297 patients. One study included an additional, separate validation cohort of 37 patients [19]. Although the combined patient population included patients with stage I–IV rectal cancer, most included cohorts were limited to stage II–III.

### 3.2. Treatment Modalities

The majority of studies employed long-course chemoradiotherapy (nCRT), with some studies involving numerous different treatment modalities such as TNT, short-course chemoradiotherapy (SCRT), chemotherapy alone, or radiotherapy alone.

### 3.3. Assessment of the Tumour Immune Microenvironment

**Figure 2:**
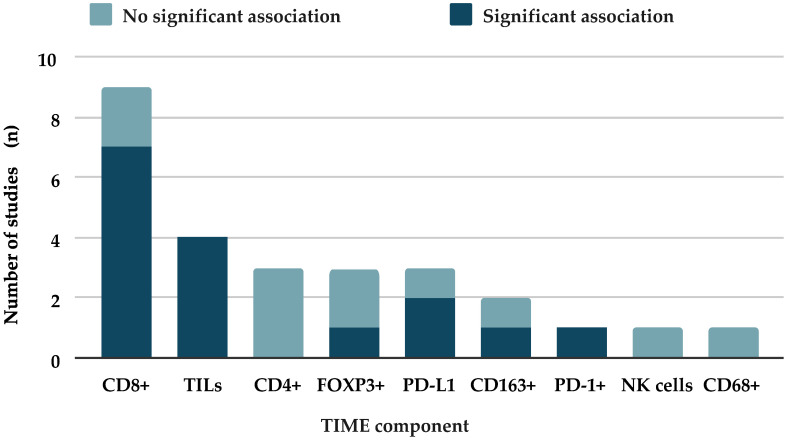
Frequency and significance of assessed biomarkers: a stacked bar chart summarises all biomarkers (x) assessed across the included studies, detailing how often each was studied (y). For each biomarker, bars were subdivided into the number of studies reporting a significant association with tumour regression versus those reporting no significant association.

The most common method of assessing pretreatment TIME components was through manual immunohistochemistry of formalin-fixed paraffin-embedded (FFPE) sections. The remaining studies employed a range of automated methods such as proteomic profiling [20] or RNA sequencing [21]. TIME components reported within studies included TILs, CD8+ cytotoxic T lymphocytes, CD4+ helper T lymphocytes, CD68+ macrophages, CD163+ macrophages, FOXP3+ regulatory T lymphocytes, PD-1+ T lymphocytes, NK (natural killer) cells, and PD-L1 (programmed death-ligand 1), with the most frequently assessed component being CD8+ (Figure 2). Conversely, several markers were only investigated within a single study. TIME components were quantified using a variety of scoring metrics (e.g., cells/mm^2^, % staining; see Table 1).

### 3.4. Measures of Tumour Regression

In all studies, the extent of tumour regression was measured by either pCR or TRG, and categorised into two distinct groups:

1. Good responders, defined as having achieved pCR, or TRG 0–2.
2. Poor responders, defined by the absence of pCR, or TRG 3–4.

**Table 1:** Characteristics of the fifteen studies included in the systematic review.

Author (Year)		Study Design	Country	Cohort Size		Range of AJCC Stages	Type of Neoadjuvant Treatment(s)	Method of Assessing Pretreatment TIME Profile		TIME Component(s) Assessed	Scoring of TIME Component	Definition of Treatment Response		
												Poor Response	Good Response	
Zhang et al., 2018 [22]		prospective cohort study	China	109		stage II–III	CRT 25–50 Gy, FOLFOX; chemotherapy alone FOLFOX	IHC on TMA sections from FFPE sections, manual assessment		CD4+ CD8+ FOXP3+ PD-L1	percentage of positively stained cells (%)	(Dworak)		
												TRG0–2	TRG3/4	
Huang et al., 2019 [23]		prospective cohort study	China	141		stage II–III	LCRT 45–55 Gy, fluoropyrimidine-based	IHC on FFPE sections, manual scoring		CD4+ CD8+	cells/mm^2^	(AJCC/UICC)		
												TRG3/4	TRG0/1	
González et al., 2020 [24]		retrospective cohort study	USA	91		stage I–III	TNT, SCRT 25 Gy, FOLFOX; SCRT 25 Gy; LCRT 50.4 Gy; chemotherapy alone; radiotherapy alone	IHC on FFPE sections, manual assessment		TILs	TILs present if ≥4 per HPF	absence of pCR	achieved pCR	
Huemer et al., 2020 [25]		retrospective cohort study	Austria, France	72		stage I–IV	LCRT 45 Gy, fluoropyrimidine-based	IHC on TMA sections from FFPE sections, manual assessment		PD-L1	cells/mm^2^	(Dworak)		
												TRG0–2	TRG3/4	
	Lai et al., 2020 [26]	retrospective cohort study	China	134	stage II–III		LCRT 45–50.4 Gy; chemotherapy alone, 5-FU or FOLFOX or FOLFOXIRI	IHC on FFPE sections, manual assessment	CD4+ CD8+		cells/mm^2^, grouped into quantiles	(AJCC)		
												TRG2/3	TRG0/1	
	Farchoukh et al., 2021 [27]	retrospective cohort study	USA	117	stage II–III		TNT, induction FOLFOX + LCRT 50.4 Gy, 5-FU-based; LCRT 50.4 Gy, 5-FU-based	IHC on FFPE sections, automated image analysis	CD8+		cells/mm^2^, grouped into high/low	(CAP TRS)		
												TRG0/1	TRG2/3	
	Sawada et al., 2021 [28]	retrospective cohort study	Japan, USA	267	stage I–III		LCRT 45 or 50.4 Gy, fluoropyrimidine-based	IHC on FFPE sections, automated scoring	CD8+		cells/mm^2^	(Dworak)		
												TRG0–2	TRG3/4	
	Xu et al., 2021 [29]	retrospective cohort study	China	210	stage II–III		LCRT 45–50 Gy, fluoropyrimidine-based; SCRT 25 Gy, FOLFOX or CAPEOX	semi- automated image analysis of H&E whole-slide images	TILs		cells/mm^2^	TRG2/3	TRG0/1	
	Yang et al., 2021 [30]	retrospective cohort study	China	76	stage II–III		LCRT 50.4 Gy, fluoropyrimidine-based	IMC and IHC on FFPE sections, manual assessment	CD8+ CD163+ FOXP3+		cells/mm^2^	absence of pCR	achieved pCR	
	Kitagawa et al., 2022 [31]	retrospective cohort study	Japan	275	stage II–III		LCRT 45/50.4 Gy, fluoropyrimidine-based	IHC on FFPE sections, manual assessment	CD8+ FOXP3+ CD68+ CD163+ PD-1+		cells/mm^2^, high/low grouped based on median values	TRG0–2	TRG3/4	
	Akiyoshi et al., 2023 [21]	retrospective case series	Japan	298	stage II–III		LCRT 45/50.4 Gy, fluoropyrimidine-based	RNA sequencing on fresh frozen biopsy samples, automated analysis	CD4+ NK		MCP-counter and ss-GSEA	TRG1/2	TRG3/4	
	Xu et al., 2023 [20]	retrospective cohort study	China	58	stage II–III		LCRT 50 Gy, fluoropyrimidine-based	PCT-PulseDIA proteomics on FFPE sections	CD8+		immunoreactive scoring, calculated by staining intensity of positive cells	TRG0/1	TRG2/3	
	Mohammed et al., 2024 [32]	prospective cohort study	Egypt	159	stage II–III		TNT, fluoropyrimidine-based + CRT with capecitabine	H&E on FFPE sections, manual assessment	TILs		International TILs Working Group guidelines (7)	absence of pCR	achieved pCR	
	Bae et al., 2025 [33]	prospective cohort study	Korea	24	stage II–III		LCRT, fluoropyrimidine-based	IHC on FFPE sections, manual assessment	PD-L1		cytoplasmic staining intensity for PD-L1: 1–4 from weak to strong	(Mandard (17))		
												TRG3–5	TRG1/2	
	Pai et al., 2025 [19]	prospective cohort study	USA	288, + 37 validation biopsies	stage I–III		TNT, FOLFOX/CAPOX /FOLFIRINOX + fluoropyrimidine-based CRT; CRT, fluoropyrimidine-based	IHC on FFPE sections; H&E-stained slides, manual assessment	TILs		cells/mm^2^	absence of pCR	achieved pCR	

Abbreviations: 5-FU, 5-fluorouracil; AJCC, American Joint Committee on Cancer; CAP TRS, College of American Pathologists Tumour Regression Grading System; CRT, chemoradiotherapy; FFPE, formalin-fixed, paraffin-embedded; FOLFOX, folinic acid and fluorouracil and oxaliplatin; FOLFOXIRI, folinic acid (leucovorin) and fluorouracil and oxaliplatin and irinotecan; FOLFIRINOX, folinic acid (leucovorin) and fluorouracil and irinotecan and oxaliplatin; H&E, Haematoxylin and Eosin; HPF, high-power field; IHC, immunohistochemistry; IMC, imaging mass cytometry; LCRT, long-course radiotherapy; MCP-counter, microenvironment cell populations-counter; nCRT, neoadjuvant chemoradiotherapy; pCR, pathological complete response; RNA, ribonucleic acid; SCRT, short-course radiotherapy; ss-GSEA, single-sample gene set enrichment analysis; TILs, tumour-infiltrating lymphocytes; TMA, tissue microarray; TNT, total neoadjuvant therapy; TRG, tumour regression grade; UICC, Union for International Cancer Control.

### 3.5. Risk of Bias Assessment

Table 2 shows the outcomes of the Quality in Prognosis Studies (QUIPS) risk of bias assessment conducted for each of the included studies. Across the papers, six domains were evaluated for bias: study participation, study attrition, predictive factor measurement, outcome measurement, study confounding, and statistical analysis and reporting. QUIPS prompting items [18] were used as criteria to determine whether a study would be high, medium, or low risk for a particular domain.

Where reported, biases often arose from high attrition, although the majority of losses were sufficiently justified by changes in the patient’s treatment journey. Bias in other studies was found due to a lack of standardised definitions for “high” vs. “low” biomarker thresholds. Studies commonly took steps to minimise bias in several ways; outcome assessors were often blinded, multivariate analyses were used to minimise the effect of confounding factors, and reproducible outcomes were used consistently across all included papers. As strengths in some domains may not compensate for weaknesses in others, scores across domains cannot be aggregated. Therefore, an overall rating was not assigned for each study, as per QUIPS recommendations [18]; however, the risk of bias was generally suggested to be low to moderate across the six domains.

### 3.6. Biomarkers Associated with Enhanced Tumour Regression

A robust biomarker is one that would be identified across multiple studies and potentially by multiple techniques. Therefore, to determine if any potential immune biomarkers might be of significance, data were compared across the different studies. Table 3 reports the correlation between the biomarkers studied and reported, and response to neoadjuvant treatment.

In total, nine articles reported data on pretreatment CD8+ T cells. Of these, seven found a statistically significant association between increased CD8+ T cell density and good response to treatment. One study in particular [21] observed a very strong positive correlation between CD8+ T cells and tumour regression (95% CI, 0.07–0.76; *p* < 0.001). TILs were assessed in four studies [19,24,29,32] with heterogeneous methods of quantification. Most studies grouped TILs using continuous measurements such as cells/mm^2^ or percentage stromal TILs [7]. In contrast, one study dichotomised TIL measurement by considering TILs present if four or more intratumoral lymphocytes were visible in a single high-power field [24]. Despite this variability, all four studies observed a statistically significant correlation between TILs and tumour regression. Three studies measured the immune checkpoint ligand, PD-L1, with two reporting a significant positive correlation with tumour regression [22,25]. Total PD-1+ cell density was also significantly associated with enhanced tumour regression (95% CI, 1.03–3.84; *p* = 0.039) but was only evaluated by one study [31].

### 3.7. Biomarkers Associated with Poor Tumour Regression

FOXP3+ Tregs were assessed by three studies and reached statistical significance only in one, where Zhang et al. strongly associated high densities with poorer treatment response [22,30,31]. Two studies measured the density of CD163+ macrophages, a subset of macrophages associated with an anti-inflammatory activity and termed an “M2 phenotype”. One study reported a statistically significant correlation between high CD163+ expression and limited tumour regression (95% CI, 0.941–0.998; *p* = 0.036) [24].

### 3.8. Other Markers

Two TIME components were assessed, which did not demonstrate any significant correlation with treatment response; these were NK cells and CD68+ macrophages (95% CI, 0.43–1.32; *p* = 0.392), which were only investigated in one study [17].

## 4. Discussion

### 4.1. Positive Predictive Biomarkers

This review of 15 studies comprising 2356 patients identified a small set of biomarkers associated with tumour regression following neoadjuvant therapy. The principal finding was the positive correlation between CD8+ density and improved response to therapy. The positive predictive potential of CD8+ has been well-documented in rectal cancer [34,35,36,37,38], although not all studies have reported this association [9]. A similar trend was observed in studies assessing TILs. Given prior findings with the immunoscore [39], it was unsurprising that all four papers studying TILs observed a statistically significant positive association with tumour regression. Although CD4+ T cells, like CD8+, are a subset of TILs, they were not significantly associated with tumour regression in any of the three studies in which they were assessed. This suggests an underlying biological mechanism specific to CD8+ TILs, rather than TILs as a whole, in accounting for their association with an enhanced therapeutic response.

### 4.2. Negative Predictive Biomarkers

In contrast, FOXP3+ regulatory cells and CD163+ macrophages were identified as TIME components associated with poorer therapeutic response, albeit within a limited number of studies and with inconsistent findings. Of the three studies measuring FOXP3+ cells, only one observed significant findings with respect to tumour regression. In this study, immune biomarkers were quantified as the percentage of positively stained cells within the assessed area and were thus influenced by the extent of infiltration of other immune cell types [22,30,31]. The other two studies quantified the number of FOXP3+ Tregs present per mm^2^, a measure less dependent on overall TIME composition. This methodological difference may account for the inconsistent findings regarding FOXP3+. Only two studies assessed CD163+ tumour-associated macrophages [30,31], yielding inconsistent results. Despite using the same treatment regimen, Yang et al. observed a negative correlation between CD163+ cells and tumour regression with a high degree of statistical confidence (95% CI, 0.941–0.998; *p* = 0.036), whilst Kitagawa et al. reported no significant correlation with response. These findings together reinforce the need for further investigation.

The findings from this review clearly present TILs—particularly the CD8+ subset—as positive predictors of response to neoadjuvant treatment, while suggesting FOXP3+ and CD163+ cells as potential negative predictive biomarkers.

Previous systematic reviews across other tumour types have investigated the pretreatment immune profile and have identified several biomarkers of predictive potential, with a high level of concordance. Among these, CD8+ was consistently associated with favourable treatment response [40,41,42], although there are a few studies reporting conflicting findings [9]. With this finding reproduced within this review, CD8+ TILs present a potential candidate immune marker for the prediction of the response to neoadjuvant therapy. Additionally, multiple systematic reviews have identified CD4+ TILs as predictors of enhanced tumour regression [40,42], despite CD4+ not reaching statistical significance across the three studies included in this review. This inconsistency may be attributed to heterogeneity in the methods of TIL quantification or the criteria used to define treatment response.

Mirroring the findings within this review, the current evidence base for FOXP3+ comprises studies observing a significant negative correlation with tumour regression [14], counterbalanced by a comparable number of studies reporting no association [9]. This highlights the need for a more focused synthesis of data elucidating the relationship between FOXP3+ and therapeutic response.

### 4.3. Mechanistic Interpretation of Immune Correlates

As positive and negative predictors of tumour regression, respectively, it follows that CD8+ and FOXP3+ regulatory cells/CD163+ macrophages may exert antagonistic pathophysiological effects in the TIME. CD8+ T cells have been implicated in the clearance of metastatic deposits in animal models [43], and have been recognised as primary mediators of tumour cell cytotoxicity [44]. It therefore follows that high densities of this immune cell type may provide greater potential for tumour regression in the context of nCRT. As briefly mentioned, this review demonstrates a stronger association between CD8+ TILs and favourable outcomes compared to CD4+ TILs. One explanation for this may be found within their distinct roles; the principal function of CD8+ cells is to exert direct cytotoxic effects against tumour cells—a strictly anti-tumoral role—whereas some CD4+ subtypes, such as Th2 cells, have been linked to tumour survival through the release of IL-4, IL-5, and IL-13 [5]. The studies reviewed here did not differentiate between Th1, Th2, and Th17 cells, although several looked at the Treg marker FoxP3.

TAMs (CD163+) and T regulatory T cells (FOXP3+) may also exhibit their pro-tumoral effects through the suppression of cytotoxic T lymphocyte responses [45,46]. Additional functions include the promotion of angiogenesis [47], a function which, though not inherently pro-tumoral, may serve a function in the vascularisation and maintenance of tumour tissue.

Given the role of CD8+ cytotoxic T cells as key mediators of anti-tumour activity, all immunological markers studied in the articles reviewed here can play a role in the function of this crucial population, including suppression or inhibition of CD8+ T cell anti-tumour cytotoxicity by TAM and Tregs. The presence of CD8+ T cells in the TIME was already known to be highly prognostic in CRC using an immunohistochemical staining protocol known as the immunoscore. This review highlights that they are also likely to be highly predictive of response to neoadjuvant therapy in rectal cancer. Whilst the mechanisms behind CD8+ T cell-mediated anti-tumour cytotoxicity are well known, the role of other suppressive immune cells, such as TAM, in mediating the response to neoadjuvant therapy is less clear. Understanding the biological role of these cells in the TIME could aid in the identification of other key biomarkers of response.

### 4.4. Strengths and Limitations

This review has several methodological strengths; the search strategy combined broad search terms for the immune environment (e.g., “immune infiltration” and “tumour microenvironment”) with focused terms related to outcomes (e.g., “pathologic complete response” and “TRG”). The final search string was thus tailored to capture a wide range of biomarkers associated with pCR or TRG. Additionally, studies were checked against specific and detailed inclusion and exclusion criteria to yield consistent and comparable studies.

Independent of the tumour immune microenvironment, response to neoadjuvant treatment is known to vary according to treatment modality [48,49,50]. Without standardisation of treatment regimens across studies, heterogeneity in treatment modalities introduces a significant confounding variable. The scope of this review was limited to studies using standardised definitions for the measurement of treatment response, thereby facilitating meaningful cross-study comparisons of tumour regression but lacking the statistical power to compare different treatment regimes. Studies that dichotomised patients into pCR vs. non-pCR cohorts [19,24,30,32] risked missing the more subtle grades of treatment response otherwise captured in those studies reporting TRG. This oversimplification of tumour regression may fail to capture statistically significant associations with partial responses to treatment. Furthermore, restricting the search to papers using pCR or TRG meant that other important articles identifying possible biomarkers of response, such as type I interferon or T cell-inflamed gene expression profiles [51], were missed, as these used other measures of response such as changes in tumour cell density.

## 5. Conclusions

The tumour immune microenvironment in rectal cancer is a newly emerging area of interest. Findings from prior systematic reviews highlight the TIME as having significant potential to predict response to neoadjuvant therapy, with subsequent opportunities for personalised treatment and improved rates of organ preservation. However, despite this progress, the current characterisation of the TIME profile lacks the depth required for patient stratification in rectal cancer.

The appraisal of current evidence within this review contributes to the establishment of an immune profile predictive of tumour response; namely, CD8+ T-cell infiltration has been identified as a potential candidate biomarker worthy of exploration in larger powered studies. Further prospective translational studies investigating changes in the TIME throughout treatment will also provide novel and deeper insights into relevant mechanisms. This deeper understanding of the tumour immune microenvironment will be a key step towards treatment stratification and personalised therapy in locally advanced rectal cancer.

## Figures and Tables

**Table 2:** Traffic-light plot highlighting the risk of bias in each study identified within the systematic review, assessed using the Quality in Prognosis Studies (QUIPS) tool.

Author (Year)	Domains					
	Study Participation	Study Attrition	Predictive Factor Measurement	Outcome Measurement	Study Confounding	Statistical Analysis and Reporting
Zhang et al., 2018 [22]	●	●	●	●	●	●
Huang et al., 2019 [23]	●	●	●	●	●	●
González et al., 2020 [24]	●	●	●	●	●	●
Huemer et al., 2020 [25]	●	●	●	●	●	●
Lai et al., 2020 [26]	●	●	●	●	●	●
Farchoukh et al., 2021 [27]	●	●	●	●	●	●
Sawada et al., 2021 [28]	●	●	●	●	●	●
Xu et al., 2021 [29]	●	●	●	●	●	●
Yang et al., 2021 [30]	●	●	●	●	●	●
Kitagawa et al., 2022 [31]	●	●	●	●	●	●
Akiyoshi et al., 2023 [21]	●	●	●	●	●	●
Xu et al., 2023 [20]	●	●	●	●	●	●
Mohammed et al., 2024 [32]	●	●	●	●	●	●
Bae et al., 2025 [33]	●	●	●	●	●	●
Pai et al., 2025 [19]	●	●	●	●	●	●

Legend: ● = low risk. ● = medium risk.

**Table 3:** A summary of the correlation between immune cells in the TIME and response to neoadjuvant therapy: for each study in the systematic review, the correlation between expression of various immune biomarkers and treatment response is presented, along with the type of neoadjuvant therapy administered.

Author (Year)	Type of Neoadjuvant Therapy	TIME Component(s) Assessed	Correlation with Treatment Response			Statistical Assessment *p*-Value (95% CI) *
			Good Response	Poor Response	No Significant Correlation	
Zhang et al., 2018 [22]	CRT; chemotherapy alone	CD4+			CD4+	*p* = 0.068
		CD8+			CD8+	*p* = 0.126
		FOXP3		**↑** **FOXP3**		***p* < 0.001**
		PD-L1	**↑** **PD-L1**			***p* = 0.001**
Huang et al., 2019 [23]	LCRT	CD4+			CD4+	*p* = 0.055 (−0.115–0.216)
		CD8+	**↑** **CD8+**			***p* = 0.003** (0.095–0.451)
González et al., 2020 [24]	TNT; SCRT; LCRT; chemotherapy alone; radiotherapy alone	TILs	**↑** **TILs**			***p* = 0.019**
Huemer et al., 2020 [25]	LCRT	PD-L1	**↑** **PD-L1**			***p* = 0.006**
Lai et al., 2020 [26]	LCRT; chemotherapy alone	CD4+			CD4+	*p* = 0.17
		CD8+	**↑** **CD8+**			***p* < 0.001** (0.07–0.76)
Farchoukh et al., 2021 [27]	TNT; LCRT	CD8+	**↑** **CD8+**			***p* = 0.04** (1.04–6.65)
Sawada et al., 2021 [28]	LCRT	CD8+	**↑** **CD8+**			***p* = 0.002** (1.47–4.99)
Xu et al., 2021 [29]	LCRT; SCRT	TILs	**↑** ** TILs**			***p* = 0.007** (1.28–4.56)
Yang et al., 2021 [30]	LCRT	CD8+	**↑** ** CD8+**			***p* = 0.002** (1.015–1.070)
		CD163+		**↑** **CD163+**		***p* = 0.036** (0.941–0.998)
		FOXP3+			FOXP3+	*p* = 0.139 (0.949–1.007)
Kitagawa et al., 2022 [31]	LCRT	CD8+	**↑** **CD8+**			***p* = 0.01** (1.21–4.34)
		CD68+			CD68+	*p* = 0.392 (0.43–1.32)
		CD163+			CD163+	*p* = 0.89 (0.6–1.84)
		FOXP3+			FOXP3+	*p* = 0.49 (0.46–1.40)
		PD-1+	**↑** **PD-1+**			***p* = 0.039** (1.03–3.84)
Akiyoshi et al., 2023 [21]	LCRT	CD8+			CD8+	*p* = 0.46
		NK			NK	*p* = 0.10
Xu et al., 2023 [20]	LCRT	CD8+	**↑** **CD8+**			***p* = 0.009**
Mohammed et al., 2024 [32]	TNT	TILs	**↑** **TILs**			***p* = 0.003** (1.885–22.713)
Bae et al., 2025 [33]	LCRT	PD-L1			PD-L1	*p* = 0.615
Pai et al., 2025 [19]	TNT, CRT	TILs	**↑** **TILs**			***p* = 0.038** (1.02–1.10)

Legend: ↑ = higher levels. Biomarkers were organised into columns depending on reported associations with tumour regression, and coloured green (good response) or red (poor response) for further visual clarification. Definitions for “good response” and “poor response” were study-specific and are detailed in Table 1. Statistically significant *p*-values are in bold. * Confidence intervals were included in the table where reported. In studies that did not report confidence intervals, only *p*-values were recorded in the table.

## Data Availability

No new data were created or analysed in this study. Data sharing is not applicable to this article.

## Supplementary Materials

The following supporting information can be downloaded at: https://www.mdpi.com/article/10.3390/cancers18081261/s1, Table S1: PubMed search string (Date of Search: 2 June 2025); Table S2: Cochrane search string (Date of Search: 2 June 2025); Table S3: Embase via Ovid search string (Date of Search: 2 June 2025); Table S4: Excluded articles (with references): Studies excluded during title and abstract screening. File S1. PRISMA_2020_checklist.
