# Supplementary material for: The Tumour Immune Microenvironment as a Predictor of the Response to Neoadjuvant Therapy in Rectal Cancer
Source: Cancers (Basel). 2026 Apr 16;18(8):1261. doi: 10.3390/cancers18081261 (PMC13115326; doi:10.3390/cancers18081261)
Supplement: Supplementary file 1 [file cancers-18-01261-s001.zip › cancers-4233078-supplementary materials Tables S1-S4.pdf]

Table S1 – PubMed Search String (Date of Search: 2nd June 2025).

Theme of Block	Search Terms
Rectal cancer	("rectal neoplasms"[MeSH Terms] **OR** "rectal neoplasm*" **OR** "rectal cancer*" **OR** "rectal carcinoma*" **OR** "rectal adenocarcinoma*") **AND**
Neoadjuvant chemoradiotherapy or total neoadjuvant therapy	("neoadjuvant therapy"[MeSH Terms] **OR** "neoadjuvant therap*" **OR** "total neoadjuvant therap*" **OR** "induction chemotherapy"[MeSH Terms] **OR** "induction chemotherap*" **OR** "consolidation chemotherapy"[MeSH Terms] **OR** "consolidation chemotherap*") **AND**
Tumor immune microenvironment	("tumor microenvironment"[MeSH Terms] **OR** "tumor microenvironment" **OR** "lymphocytes, tumor-infiltrating"[MeSH Terms] **OR** "tumor-infiltrating lymphocyte*" **OR** "tumor immune microenvironment" **OR** "immune landscape" **OR** "immune infiltration") **AND**
Treatment response	("pathologic complete response"[MeSH Terms] **OR** "pathologic complete response" **OR** "tumor regression grade")

Table S2. – Cochrane Search String (Date of Search: 2nd June 2025).

Line	Search term	Results
#1	rectal NEXT cancer*:ti,ab,kw	4341
#2	rectal NEXT carcinoma*:ti,ab,kw	381
#3	rectal NEXT neoplasm*:ti,ab,kw	2817
#4	rectal NEXT adenocarcinoma*:ti,ab,kw	458
#5	MeSH descriptor: [Rectal Neoplasms] explode all trees	2957
#6	#1 OR #2 OR #3 OR #4 OR #5	8194
#7	neoadjuvant NEXT therap*:ti,ab,kw	6188
#8	MeSH descriptor [Neoadjuvant Therapy] explode all trees	2767
#9	"chemoradiotherapy":ti,ab,kw	9473
#10	"total neoadjuvant therapy":ti,ab,kw	171
#11	MeSH descriptor: [Induction Chemotherapy] explode all trees	682
#12	induction NEXT chemotherap*:ti,ab,kw	3905
#13	MeSH descriptor: [Consolidation Chemotherapy] explode all trees	169
#14	consolidation NEXT chemotherap*:ti,ab,kw	905
#15	#7 OR #8 OR #9 OR #10 OR #11 OR #12 OR #13 OR #14	17849
#16	MeSH descriptor [Tumor Microenvironment] explode all trees	210
#17	"tumor microenvironment":ti,ab,kw	1186
#18	MeSH descriptor: [Lymphocytes, Tumor-Infiltrating] explode all trees	216
#19	tumor-infiltrating NEXT lymphocyte*:ti,ab,kw	641
#20	"tumor immune microenvironment":ti,ab,kw	128
#21	"immune landscape":ti,ab,kw	42
#22	"immune infiltration":ti,ab,kw	111

#23	#16 OR #17 OR #18 OR #19 OR #20 OR #21 OR #22	1910
#24	MeSH descriptor: [Pathologic Complete Response] explode all trees	24
#25	“pathologic complete response”:ti,ab,kw	1251
#26	“tumor regression grade”:ti,ab,kw	190
#27	#24 OR #25 OR #26	1422
#28	#6 AND #15 AND #23 AND #27	5

Table S3. – Embase via Ovid Search String (Date of Search: 2nd June 2025).

Line	Search term	Results
#1	exp rectum tumor/	87861
#2	exp rectal cancer/	66386
#3	exp rectal carcinoma/	18523
#4	exp rectal adenocarcinoma/	2692
#5	1 or 2 or 3 or 4	87861
#6	"neoadjuvant therapy".ti,ab,kw.	25078
#7	"chemoradiotherapy".ti,ab,kw.	46213
#8	"total neoadjuvant therapy".mp.	1112
#9	exp induction chemotherapy/	21828
#10	exp consolidation chemotherapy/	5711
#11	6 or 7 or 8 or 9 or 10	90703
#12	exp tumor microenvironment/	14446
#13	exp tumor infiltrating lymphocyte/	50607
#14	"tumor immune microenvironment".ti,ab,kw.	8752
#15	"immune landscape".ti,ab,kw.	4295
#16	"immune infiltration".ti,ab,kw.	14118
#17	11 or 12 or 13 or 14 or 15	186734
#18	exp pathologic complete response/	4854
#19	"tumor regression grade".ti,ab,kw.	1721
#20	18 or 19	6500
#21	5 and 11 and 17 and 20	48

Table S4 – Excluded articles (with references)

Studies excluded during title & abstract screening

Study (First author, year)	Title	Reason for Exclusion
Spolverato et al, 2021	Immunological microenvironment in rectal adenocarcinoma treatment. Predictors of sustained complete response after neoadjuvant chemo/radiotherapy for locally advanced rectal cancer	Not a primary research article
Mast et al., 2024	Exentro: Exercise during neoadjuvant chemoradiation treatment to improve rectal and esophageal cancer outcome	Wrong population (included oesophageal cancer)
Aday et al., 2022	Investigation into the effect of neoadjuvant therapy and tumor regression grade on the shrinkage of distal surgical margin in rectal cancer: A prospective case-control study	Wrong population (subcohort did not receive neoadjuvant therapy)
Alderdice et al., 2017	Natural killer-like signature observed post therapy in locally advanced rectal cancer is a determinant of pathological response and improved survival	No analysis of relationship between pretreatment TIME and treatment response
Badon et al., 2023	Carbonic Anhydrase IX Expression and Treatment Response Measured in Rectal Adenocarcinoma Following Neoadjuvant Chemo-Radiotherapy	No analysis of pretreatment TIME
Bando et al., 2022	Preoperative Chemoradiotherapy plus Nivolumab before Surgery in Patients with Microsatellite Stable and Microsatellite Instability-High Locally Advanced Rectal Cancer.	Patients received concomitant biologic therapy
Capdevila et al., 2020	Phase II study of durvalumab plus total neoadjuvant therapy (TNT) in locally advanced rectal cancer: The GEMCAD-1703 DUREC trial	Patients received concomitant biologic therapy
Chen et al., 2019	Prognostic relevance of programmed cell death-ligand 1 expression and CD8+ TILs in rectal cancer patients before and after neoadjuvant chemoradiotherapy	No analysis of relationship between pretreatment TIME and treatment response
Chen et al., 2024	Analysis of tumor microenvironment alterations in partially responsive rectal cancer patients treated with neoadjuvant chemoradiotherapy	No analysis of relationship between pretreatment TIME and treatment response
Chiang et al., 2025	Bevacizumab Enhances Pathological Complete Response and Survival When Added to Neoadjuvant Chemoradiotherapy in Pd-L1-Negative Tumor Microenvironment of Locally Advanced Rectal Cancer	Patients received concomitant biologic therapy
Corro et al., 2025	Integrative analysis of transcriptomic data reveals a predictive gene signature for	No analysis of pretreatment TIME

	chemoradiotherapy response in rectal cancer	
deMoraes et al., 2024	Impact of adjuvant chemotherapy on survival after pathological complete response in rectal cancer: a meta-analysis of 31,558 patients	Not a primary research article
Felder et al., 2024	Which treatment for which patient: rectal cancer management after PROSPECT Trial	Not a primary research article
Gnetti et al., 2021	Influence of preoperative chemoradiation on tumor-infiltrating lymphocytes in locally advanced rectal cancer: the STAR-01 cohort	No analysis of relationship between pretreatment TIME and treatment response
Goedegebuure et al., 2021	Pre-treatment tumor-infiltrating T cells influence response to neoadjuvant chemoradiotherapy in esophageal adenocarcinoma	Wrong population (oesophageal carcinoma)
Gögenur et al., 2023	Neoadjuvant intratumoral influenza vaccine treatment in patients with proficient mismatch repair colorectal cancer leads to increased tumor infiltration of CD8+ T cells and upregulation of PD-L1: a phase 1/2 clinical trial.	Wrong population (included colon cancer)
Hillson et al., 2024	Radiation-induced changes in gene expression in rectal cancer specimens	Not a primary research article
Imai et al., 2024	Correlation of enriched specific subset of immune cells nearby tumor associated macrophage (TAM) with pathologic complete response (pCR) of concurrent chemoradiotherapy followed by nivolumab in locally advanced rectal cancer (LARC)	Patients received concomitant biologic therapy
Imaizumi et al., 2017	Immunological features of resected tumor after neoadjuvant chemotherapy (NAC) and chemoradiotherapy (CRT) become the superior prediction markers for recurrence in rectal cancer	No analysis of relationship between pretreatment TIME and treatment response
Inamori et al., 2021	Translational research of voltage-A1: Efficacy predictors of preoperative chemoradiotherapy and consolidation nivolumab inpatients with both microsatellite stable and microsatellite instability-high locally advanced rectal cancer	Patients received concomitant biologic therapy
Inamori et al., 2021	P25-4 Efficacy predictors of preoperative CRT and consolidation nivolumab in patients with locally advanced rectal cancer	Patients received concomitant biologic therapy
Jameson et al., 2022	TNT: Raising more questions than answers?	Not a primary research article

Ji et al., 2019	Advances in the prediction of the efficacy and sensitivity of neoadjuvant chemoradiotherapy in rectal cancer.	No full text available
Kong et al., 2019	Prognostic impact of tumor-infiltrating lymphocytes in primary and metastatic colorectal cancer: A systematic review and meta-Analysis	Not a primary research article
Lee et al., 2022	Biomarkers of favorable vs. unfavorable responses in locally advanced rectal cancer patients receiving neoadjuvant concurrent chemoradiotherapy	Not a primary research article
Li et al., 2024	Are the tumor microenvironment characteristics of pretreatment biopsy specimens of colorectal cancer really effectively predict the efficacy of neoadjuvant therapy: A retrospective multicenter study.	No analysis of relationship between pretreatment TIME and treatment response
He et al., 2023	Genomic features, evolutionary patterns, and minimal residual disease at surgical margins as novel prognostic/predictive biomarkers in locally advanced rectal cancer	No full text available
Liu et al., 2019	Lymphocyte nadir predicts tumor response and survival in locally advanced rectal cancer after neoadjuvant chemoradiotherapy: Immunologic relevance.	No pretreatment TIME analysis
Liu et al., 2020	Different immune profiling alterations of tumor microenvironment in locally advanced rectal cancer following short-course radiotherapy and long-course chemoradiotherapy	No analysis of relationship between pretreatment TIME and treatment response
Li et al., 2024	Rectal adenocarcinoma: Ex vivo 9.4T MRI-correlation with histopathologic treatment response to neoadjuvant chemoradiotherapy	No pretreatment TIME analysis
Miyakita et al., 2020	Changes in tumor-infiltrating lymphocytes and inflammatory blood factors during chemoradiation therapy in rectal cancer	No pretreatment TIME analysis
Negri et al., 2020	Preoperative chemoradiotherapy of locally advanced rectal cancer and tumor-infiltrating lymphocyte response related: The smart-star study	Wrong population (not specific to rectal cancer)
Pei et al., 2024	PD-1 blockade enhances the effect of targeted chemotherapy on locally advanced pMMR/MSS colorectal cancer.	Wrong population (colon cancer)
Peng et al., 2023	Develop and validate a radiomics space-time model to predict the pathological complete response in patients undergoing neoadjuvant treatment of rectal cancer: an	No analysis of relationship between pretreatment TIME and treatment response

	artificial intelligence model study based on machine learning	
Polack et al., 2025	The tumour-stroma ratio as predictive aid towards a biopsy-based treatment strategy in rectal carcinoma	No pretreatment TIME analysis
Salvatore et al., 2021	Phase II study of preoperative chemoradiotherapy plus avelumab in patients with locally advanced rectal cancer	Patients received concomitant biologic therapy
Sanford et al., 2021	Innate: immunotherapy during neoadjuvant therapy for rectal cancer to elucidate local and systemic therapeutic responses	Patients received concomitant immunotherapy
Serna et al., 2020	Fusobacterium nucleatum persistence and risk of recurrence after preoperative treatment in locally advanced rectal cancer.	No analysis of relationship between pretreatment TIME and treatment response
Shamseddine et al., 2020	Short-course radiation followed by mFOLFOX-6 plus avelumab for locally-advanced rectal adenocarcinoma	Patients received concomitant biologic therapy
Yu et al., 2024	Tumor regression and safe distance of distal margin after neoadjuvant therapy for rectal cancer	Not a primary research article
Yunlong et al., 2023	The efficiency of neoadjuvant chemotherapy in colon cancer with mismatch repair deficiency	Wrong population (colon cancer)

Studies excluded during full-text screening

Study (First author, year)	Title	Reason for Exclusion
Chen et al., 2020	The Effects of Neoadjuvant Treatment on the Tumor Microenvironment in Rectal Cancer: Implications for Immune Activation and Therapy Response	No analysis of pretreatment TIME
Lafarge et al., 2024	Image-based consensus molecular subtyping in rectal cancer biopsies and response to neoadjuvant chemoradiotherapy	No analysis of relationship between pretreatment TIME and treatment response
Lim et al., 2014	Effect of neoadjuvant chemoradiation on tumor-infiltrating/associated lymphocytes in locally advanced rectal cancers	No analysis of relationship between pretreatment TIME and treatment response
Miyakita et al., 2025	Changes in tumor-infiltrating lymphocytes and inflammatory blood factors during chemoradiation therapy in rectal cancer	No analysis of relationship between pretreatment TIME and treatment response
McCoy et al., 2016	Low stromal Foxp3+ regulatory T-cell density is associated with complete response to neoadjuvant chemoradiotherapy in rectal cancer	No pretreatment TIME analysis
Negri et al., 2020	Impact of preoperative chemoradiotherapy on tumor infiltrating lymphocytes in locally	No analysis of relationship between pretreatment TIME and treatment response

	advanced rectal cancer: The SMART-STAR study	
Negri et al., 2021	Influence of preoperative chemo-radiation on tumor-infiltrating lymphocytes in locally advanced rectal cancer and tumor response: results from the star-01 cohort	Not a primary research article
Song et al., 2019	Predictive and prognostic relevance of CD8+ tumor-infiltrating lymphocyte density combined with PD-L1 expression in locally advanced rectal cancer patients receiving preoperative chemoradiotherapy	Not a primary research article
Walden et al., 2024	Utilizing quantitative pathologic analysis of digitized images of rectal cancer (RC) to predict response to neoadjuvant therapy (NAT)	Not a primary research article

Aday U, Kiliçarslan A, Böyük A, Akkoç H. Investigation into the effect of neoadjuvant therapy and tumor regression grade on the shrinkage of distal surgical margin in rectal cancer: A prospective case-control study. Indian J Pathol Microbiol. 2022;65(2). doi:10.4103/IJPM.IJPM_1130_20

Alderdice M, Dunne PD, Cole AJ, O'Reilly PG, McArt DG, Bingham V, Fuchs MA, McQuaid S, Loughrey MB, Murray GI, et al. Natural killer-like signature observed post therapy in locally advanced rectal cancer is a determinant of pathological response and improved survival. Modern Pathology. 2017;30(9). doi:10.1038/modpathol.2017.47

Bádon ES, Beke L, Mokánszki A, András C, Méhes G. Carbonic Anhydrase IX Expression and Treatment Response Measured in Rectal Adenocarcinoma Following Neoadjuvant Chemo-Radiotherapy. Int J Mol Sci. 2023;24(3). doi:10.3390/ijms24032581

Bando H, Tsukada Y, Inamori K, Togashi Y, Koyama S, Kotani D, Fukuoka S, Yuki S, Komatsu Y, Homma S, et al. Preoperative Chemoradiotherapy plus Nivolumab before Surgery in Patients with Microsatellite Stable and Microsatellite Instability–High Locally Advanced Rectal Cancer. Clinical Cancer Research. 2022;28(6). doi:10.1158/1078-0432.CCR-21-3213

Capdevila J, Macias Declara I, Riesco Martinez MC, Maurel J, Hernando J, Alonso V, Graña Suárez B, Gallego Plazas J, Losa F, Vera R, et al. Phase II study of durvalumab plus total neoadjuvant therapy (TNT) in locally advanced rectal cancer: The GEMCAD-1703 DUREC trial. Journal of Clinical Oncology. 2020;38(15_suppl). doi:10.1200/jco.2020.38.15_suppl.tps4122

Chen CC, Wu ML, Huang KC, Huang IP, Chung YL. The Effects of Neoadjuvant Treatment on the Tumor Microenvironment in Rectal Cancer: Implications for Immune Activation and Therapy Response. Clin Colorectal Cancer. 2020;19(4). doi:10.1016/j.clcc.2020.04.002

Chen H, Zhang JH, Hao Q, Wu XL, Guo JX, Huang CX, Zhang J, Xing GS, An ZL, Ling Y, et al. Analysis of tumor microenvironment alterations in partially responsive rectal cancer patients treated with neoadjuvant chemoradiotherapy. Int J Colorectal Dis. 2024;39(1). doi:10.1007/s00384-024-04672-1

Chiang Y, Liang JT, Shun CT, Chen YH, Tsai CL, Hung JS, Huang J, Chen TC, Tsai CH, Cheng JCH. Bevacizumab Enhances Pathological Complete Response and Survival When Added to

Neoadjuvant Chemoradiotherapy in Pd-L1-Negative Tumor Microenvironment of Locally Advanced Rectal Cancer. 2025. doi:10.2139/ssrn.5191851

Corrò C, Carvalho JVM, Rapti M, Angelino P, Tihy M, Bakaric A, Puppa G, Wirapati P, Durham A, Ris F, et al. Integrative analysis of transcriptomic data reveals a predictive gene signature for chemoradiotherapy response in rectal cancer. iScience. 2026;29(1). doi:10.1016/j.isci.2025.114455

de Moraes FCA, Kelly FA, Souza MEC, Burbano RMR. Impact of adjuvant chemotherapy on survival after pathological complete response in rectal cancer: a meta-analysis of 31,558 patients. Int J Colorectal Dis. 2024;39(1). doi:10.1007/s00384-024-04668-x

Felder S, Frakes J, George M, Rosen A, Sahin IH. Which Treatment for Which Patient: Rectal Cancer Management After PROSPECT Trial. Oncology (United States). 2024. doi:10.46883/2024.25921019

Goedegebuure RSA, Harrasser M, de Klerk LK, van Schooten TS, van Grieken NCT, Eken M, Grifhorst MS, Pocorni N, Jordanova ES, van Berge Henegouwen MI, et al. Pre-treatment tumor-infiltrating T cells influence response to neoadjuvant chemoradiotherapy in esophageal adenocarcinoma. Oncoimmunology. 2021;10(1). doi:10.1080/2162402X.2021.1954807

Gögenur M, Balsevicius L, Bulut M, Colak N, Justesen TF, Fiehn AMK, Jensen MB, Høst-Rasmussen K, Cappelen B, Gaggar S, et al. Neoadjuvant intratumoral influenza vaccine treatment in patients with proficient mismatch repair colorectal cancer leads to increased tumor infiltration of CD8+ T cells and upregulation of PD-L1: a phase 1/2 clinical trial. J Immunother Cancer. 2023;11(5). doi:10.1136/jitc-2023-006774

He K, Li L, Li A, Xu Y, Pang J, Mu D, Ma J, Ge H, Maleki A, Qin X, et al. Genomic features, evolutionary patterns and minimal residual disease at surgical margins as novel prognostic/predictive biomarkers in locally advanced rectal cancer. Clin Transl Med. 2023;13(6). doi:10.1002/ctm2.1286
Hillson LVS, McCulloch AK, Edwards J, Dunne PD, O'Cathail SM, Roxburgh CS. Radiation-induced changes in gene expression in rectal cancer specimens. Clinical and Translational Oncology. 2024;26(6). doi:10.1007/s12094-023-03361-9

Imai M, Bando H, Tsukada Y, Inamori K, Kato T, Komatsu Y, Uemura M, Yuki S, Taketomi A, Fujisawa T, et al. Correlation of enriched specific subset of immune cells nearby tumor associated macrophage (TAM) with pathologic complete response (pCR) of concurrent chemoradiotherapy followed by nivolumab in locally advanced rectal cancer (LARC). Journal of Clinical Oncology. 2024;42(23_suppl). doi:10.1200/jco.2024.42.23_suppl.58

Imaizumi K, Suzuki T, Shimomura M, Tsukada Y, Sasaki T, Nishizawa Y, Kojima M, Ito M, Nakatsura T. Immunological features of resected tumor after neoadjuvant chemotherapy (NAC) and chemoradiotherapy (CRT) become the superior prediction markers for recurrence in rectal cancer. Annals of Oncology. 2017;28. doi:10.1093/annonc/mdx393.035

Inamori K, Togashi Y, Bando H, Tsukada Y, Fukuoka S, Suzuki A, Suzuki Y, Kotani D, Kojima M, Fukui M, et al. P25-4 Efficacy predictors of preoperative CRT and consolidation nivolumab in patients with locally advanced rectal cancer. Annals of Oncology. 2021;32. doi:10.1016/j.annonc.2021.05.724

Inamori K, Togashi Y, Bando H, Tsukada Y, Suzuki A, Suzuki Y, Kotani D, Fukuoka S, Kojima M, Fukui M, et al. Translational research of voltage-A1: Efficacy predictors of preoperative chemoradiotherapy and subsequent nivolumab monotherapy in patients with microsatellite-stable locally advanced rectal cancer. Journal of Clinical Oncology. 2020;38(15_suppl). doi:10.1200/jco.2020.38.15_suppl.4073

Jameson MB, Stevenson ARL, Ngan SY. TNT: Raising more questions than answers? Asia-Pacific Journal of Clinical Oncology. 2022. doi:10.1111/ajco.13760

Ji Q, Li Y, Wu T. Advances in the prediction of the efficacy and sensitivity of neoadjuvant chemoradiotherapy in rectal cancer. Chinese Journal of Gastrointestinal Surgery / Zhonghua Wei Chang Wai Ke Za Zhi. 2019. doi:10.3760/cma.j.issn.1671-0274.2019.04.015

Kong JC, Guerra GR, Pham T, Mitchell C, Lynch AC, Warrier SK, Ramsay RG, Heriot AG. Prognostic impact of tumor-infiltrating lymphocytes in primary and metastatic colorectal cancer: A systematic review and meta-Analysis. Diseases of the Colon and Rectum. 2019. doi:10.1097/DCR.0000000000001332

Lafarge MW, Domingo E, Sirinukunwattana K, Wood R, Samuel L, Murray G, Richman SD, Blake A, Sebag-Montefiore D, Gollins S, et al. Image-based consensus molecular subtyping in rectal cancer biopsies and response to neoadjuvant chemoradiotherapy. NPJ Precis Oncol. 2024;8(1). doi:10.1038/s41698-024-00580-3

Lee HH, Chen CH, Huang YH, Chiang CH, Huang MY. Biomarkers of Favorable vs. Unfavorable Responses in Locally Advanced Rectal Cancer Patients Receiving Neoadjuvant Concurrent Chemoradiotherapy. Cells. 2022. doi:10.3390/cells11101611

Li B, Chen L, Huang Y, Wu M, Fang W, Zou X, Zheng L, Xiao Q. Are the tumor microenvironment characteristics of pretreatment biopsy specimens of colorectal cancer really effectively predict the efficacy of neoadjuvant therapy A retrospective multicenter study. Medicine (United States). 2024;103(35). doi:10.1097/MD.0000000000039429

Li Z, Yuan Y, Liu M, Bo T, Ma X, Wang H, Chen C, Shi X, Hao W, Bai C, et al. Rectal adenocarcinoma: Ex vivo 9.4T MRI—correlation with histopathologic treatment response to neoadjuvant chemoradiotherapy. Cancer Med. 2024;13(15). doi:10.1002/cam4.70075

Lim SHS, Chua W, Cheng C, Descallar J, Ng W, Solomon M, Bokey L, Wong K, Lee MT, de Souza P, et al. Effect of neoadjuvant chemoradiation on tumor-infiltrating/associated lymphocytes in locally advanced rectal cancers. Anticancer Res. 2014;34(11).

Liu H, Wang H, Wu J, Wang Y, Zhao L, Li G, Zhou M. Lymphocyte nadir predicts tumor response and survival in locally advanced rectal cancer after neoadjuvant chemoradiotherapy: Immunologic relevance. Radiotherapy and Oncology. 2019;131. doi:10.1016/j.radonc.2018.12.001

Liu N, Wu X, Deng X, Wang Z, Jiang D, Yang L, Zhang H, Wang X. Different Immune Profiling Alterations of Tumor Microenvironment in Locally Advanced Rectal Cancer Following Short-Course Radiotherapy and Long-Course Chemoradiotherapy. International Journal of Radiation Oncology*Biology*Physics. 2020;108(3). doi:10.1016/j.ijrobp.2020.07.1718

Mast I, Gootjes E, Rütten H, den Hartogh M, Nagtegaal I, van den Heuvel B, van den Heuvel B, Rosman C, Klarenbeek B, Hopman MF, de Wilt H, et al. Exercise During Neoadjuvant

Chemoradiation Treatment To Improve Rectal And Esophageal Cancer Outcome (EXENTRO) Pilot Trial. Med Sci Sports Exerc. 2024;56(10S). doi:10.1249/01.mss.0001057356.60038.b7

McCoy MJ, Hemmings C, Miller TJ, Austin SJ, Bulsara MK, Zeps N, Nowak AK, Lake RA, Platell CF. Low stromal Foxp3+ regulatory T-cell density is associated with complete response to neoadjuvant chemoradiotherapy in rectal cancer. Br J Cancer. 2015;113(12). doi:10.1038/bjc.2015.427

Miyakita H, Ogimi T, Kayano H, Mori M, Yamamoto S. Changes in Tumor-Infiltrating Lymphocytes and Inflammatory Blood Factors during Chemoradiation Therapy in Rectal Cancer. Oncology (Switzerland). 2025. doi:10.1159/000545312

Negri F, Bottarelli L, Gnetti L, Campanini N, Negru E, Bergamo F, Siena S, Frisinghelli M, Petric M, Chiaulon G, et al. Preoperative chemoradiotherapy of locally advanced rectal cancer and tumor-infiltrating lymphocyte response related: The smart-star study. Tumori. 2020;106(2 SUPPL).

Negri F, Gnetti L, Bottarelli L, Campanini N, Bergamo F, Siena S, Frisinghelli M, Petric M, Chiaulon G, Mosconi S, et al. 426P Impact of preoperative chemoradiotherapy on tumor infiltrating lymphocytes in locally advanced rectal cancer: The SMART-STAR study. Annals of Oncology. 2020;31. doi:10.1016/j.annonc.2020.08.537

Negri F, Gnetti L, Bottarelli L, Campanini N, Negru ME, Bergamo F, Frisinghelli M, Chiaulon G, Tagliagambe A, Morabito A, et al. Influence of preoperative chemoradiation on tumor-infiltrating lymphocytes in locally advanced rectal cancer: The STAR-01 cohort. Journal of Clinical Oncology. 2021;39(15_suppl). doi:10.1200/jco.2021.39.15_suppl.3611

Pei F, He W, Duan Y, Yao Q, Zhao Y, Fan X, Liu S, Chen H, He F, Liu T, et al. PD-1 blockade enhances the effect of targeted chemotherapy on locally advanced pMMR/MSS colorectal cancer. Cancer Med. 2024;13(12). doi:10.1002/cam4.7224

Peng J, Wang W, Jin H, Qin X, Hou J, Yang Z, Shu Z. Develop and validate a radiomics space-time model to predict the pathological complete response in patients undergoing neoadjuvant treatment of rectal cancer: an artificial intelligence model study based on machine learning. BMC Cancer. 2023;23(1). doi:10.1186/s12885-023-10855-w

Polack M, van Pelt GW, van den Heuvel DH, Klein-Kranenbarg EM, Roodvoets AGH, Putter H, Crobach ASL, Nagtegaal ID, Peeters KCMJ, Tollenaar RAE, et al. The tumour–stroma ratio as predictive aid towards a biopsy-based treatment strategy in rectal carcinoma. Histopathology. 2025;87(1). doi:10.1111/his.15423

Salvatore L, Bensi M, Corallo S, Bergamo F, Pellegrini I, Rasola C, Borelli B, Tamburini E, Randon G, Galuppo S, et al. O-12 Phase II study of preoperative chemoradiotherapy plus avelumab in patients with locally advanced rectal cancer: The AVANA study. Annals of Oncology. 2021;32. doi:10.1016/j.annonc.2021.05.016

Sanford N, Elghonaimy E, Kardosh A, Kazmi S, Pogacnik JS, Yang X, Timmerman R, Aguilera T. 411 INNATE: immunotherapy during neoadjuvant therapy for rectal cancer to elucidate local and systemic therapeutic responses. In. 2021. doi:10.1136/jitc-2021-sitc2021.411

Serna G, Ruiz-Pace F, Hernando J, Alonso L, Fasani R, Landolfi S, Comas R, Jimenez J, Elez E, Bullman S, et al. Fusobacterium nucleatum persistence and risk of recurrence after preoperative treatment in locally advanced rectal cancer. Annals of Oncology. 2020;31(10). doi:10.1016/j.annonc.2020.06.003

Shamseddine A, Zeidan Y, Khalifeh IM, Kattan JG, Turfa R, Mukherji D, Temraz SN, Jamali F, Shaib YH, Soweid A, et al. Short-course radiation followed by mFOLFOX-6 plus avelumab for locally-advanced rectal adenocarcinoma. BMC Cancer. 2020;20(1). doi:10.1186/s12885-020-07333-y

Song C, Kwak Y, Lee HS, Kang SB, Kim JS. Predictive and prognostic relevance of CD8+ tumor-infiltrating lymphocyte density combined with PD-L1 expression in locally advanced rectal cancer patients receiving preoperative chemoradiotherapy. J Immunother Cancer. 2019;7.

Spolverato G, Fassan M, Chiminazzo V, Businello G, Angriman I, Kotsafti A, Ruffolo C, Marchegiani F, Vignotto C, Pozza A, et al. Immunological microenvironment in rectal Adenocarcinoma Treatment (IMMUNOREACT 2; NCT04917263): preliminary results on prediction of complete response to neoadjuvant therapy in rectal cancer. European Journal of Surgical Oncology. 2022;48(2). doi:10.1016/j.ejso.2021.12.185

Walden D, Eslinger C, Emiloju OE, Storandt MH, Hagen CE, Pfeiffer A, Sonbol BB, Ahn DH, Bekaii-Saab TS, Ness A, et al. Utilizing quantitative pathologic analysis of digitized images of rectal cancer (RC) to predict response to neoadjuvant therapy (NAT). Journal of Clinical Oncology. 2024;42(3). doi:10.1200/JCO.2024.42.3_suppl.200

Yu G, Chi H, Zhao G, Wang Y. Tumor regression and safe distance of distal margin after neoadjuvant therapy for rectal cancer. Frontiers in Oncology. 2024. doi:10.3389/fonc.2024.1375334

Yunlong W, Tongtong L, Hua Z. The efficiency of neoadjuvant chemotherapy in colon cancer with mismatch repair deficiency. Cancer Med. 2023;12(3). doi:10.1002/cam4.5076
